# FUCHS—towards full circular RNA characterization using RNAseq

**DOI:** 10.7717/peerj.2934

**Published:** 2017-02-28

**Authors:** Franziska Metge, Lisa F. Czaja-Hasse, Richard Reinhardt, Chistoph Dieterich

**Affiliations:** 1 Max-Planck Institute for Biology of Ageing, Cologne, Germany; 2 Max-Planck-Genome-Centre Cologne, Cologne, Germany; 3 Department of Internal Medicine III and Klaus Tschira Institute for Integrative Computational Cardiology, University Hospital Heidelberg, Germany; 4 Partner site Heidelberg/Mannheim, German Centre for Cardiovascular Research (DZHK), Heidelberg, Germany

**Keywords:** Circular RNA, Alternative-splicing, Transcriptomics

## Abstract

Circular RNAs (circRNAs) belong to a recently re-discovered species of RNA that emerge during RNA maturation through a process called back-splicing. A downstream 5′ splice site is linked to an upstream 3′ splice site to form a circular transcript instead of a canonical linear transcript. Recent advances in next-generation sequencing (NGS) have brought circRNAs back into the focus of many scientists. Since then, several studies reported that circRNAs are differentially expressed across tissue types and developmental stages, implying that they are actively regulated and not merely a by-product of splicing. Though functional studies have shown that some circRNAs could act as miRNA-sponges, the function of most circRNAs remains unknown. To expand our understanding of possible roles of circular RNAs, we propose a new pipeline that could fully characterizes candidate circRNA structure from RNAseq data—FUCHS: **FU**ll **CH**aracterization of circular RNA using RNA-**S**equencing. Currently, most computational prediction pipelines use back-spliced reads to identify circular RNAs. FUCHS extends this concept by considering all RNA-seq information from long reads (typically >150 bp) to learn more about the exon coverage, the number of double break point fragments, the different circular isoforms arising from one host-gene, and the alternatively spliced exons within the same circRNA boundaries. This new knowledge will enable the user to carry out differential motif enrichment and miRNA seed analysis to determine potential regulators during circRNA biogenesis. FUCHS is an easy-to-use Python based pipeline that contributes a new aspect to the circRNA research.

## Introduction

Circular RNAs (circRNAs) emerge during RNA maturation by a process called back-splicing. A downstream 5′ splice site is linked to an upstream 3′ splice site to form a circular transcript instead of a canonical linear transcript. CircRNAs were discovered in the early nineties (Nigro et al., 1991; Capel et al., 1993), and dismissed as non-functional by-products of splicing. Recent advances in next-generation sequencing (NGS) brought circRNAs back into the focus of many scientists as short reads, which cover back-splicing junctions. These are well represented in rRNA-depleted RNA-seq samples.

Since then, several studies reported that circRNAs are differentially expressed across tissue types and developmental stages, implying that circRNAs are regulated and not merely a by-product of splicing (Salzman et al., 2013; You et al., 2015). In fact, Ivanov et al. (2015) showed that regions, which flank circRNA loci, are enriched for short reverse complementary matches (RCM) that drive circularization events.

The current working model predicts that RCMs (especially Alu repeats) are a target of differential RNA A → I editing. An increase in RNA editing “melts” the corresponding intermediate hairpin structures by lowering base pairing energies, which leads to a decrease in circRNA production. Furthermore, Liang & Wilusz (2014) proved that only short sub-sequences of Alu repeats in the flanking introns of the human ZKSCAN1 gene are necessary to circularize exon 2 and 3 of ZKSCAN1. This mechanism seems to be sufficient to drive circularization. When other exons (from a different gene) were cloned in between these Alu repeats they also circularized.

Conn et al. (2015) reported that the RNA binding protein Quaking positively regulates circularization. They demonstrated that changes in the expression of Quaking affects exon circularization in a model of epithelial-mesenchymal transition (EMT). Quaking binds to adjacent motifs in the flanking introns. Knocking down Quaking as well as mutating the Quaking binding motifs significantly decreases the expression of the respective circRNA.

Functional circRNA studies are scarce to date, but suggest that some circRNAs could act as miRNA-sponges (Hansen et al., 2013; Memczak et al., 2013). Nevertheless, the function of most circRNAs remains unknown. Since some circRNAs are stable, detectable in peripheral blood, and show differential abundance patterns in control vs. disease conditions, they have been proposed as new biomarkers. For example, hsa_circ_001988 has been shown to correlate with colorectal cancer (Wang et al., 2015) while hsa_circ_101222 has been associated with pre-eclampsia (Zhang et al., 2016b).

To expand our understanding of possible roles of circular RNAs, we propose a new pipeline that aims to fully characterize candidate circRNA sequence and structure from RNAseq data—FUCHS: **FU**ll **CH**aracterization of circular RNA using RNA-**S**equencing. Currently, most computational prediction pipelines only use RNA-seq reads to identify back-splicing events. FUCHS extends this concept by considering all RNA-seq information from long reads (typically >150 bp).

By running FUCHS, the user will learn more about the exon coverage, the amount of double break point fragments, the different circular isoforms arising from one host-gene, and the alternatively spliced exons within the same circRNA. This new knowledge will enable the user to perform differential motif enrichment and miRNA seed analysis to determine potential regulators during circRNA biogenesis, as well as pathways circRNAs may play a role in. FUCHS is an easy-to-use Python based pipeline that contributes with new aspects to the field of circRNA research.

## Methods

**Figure 1:**
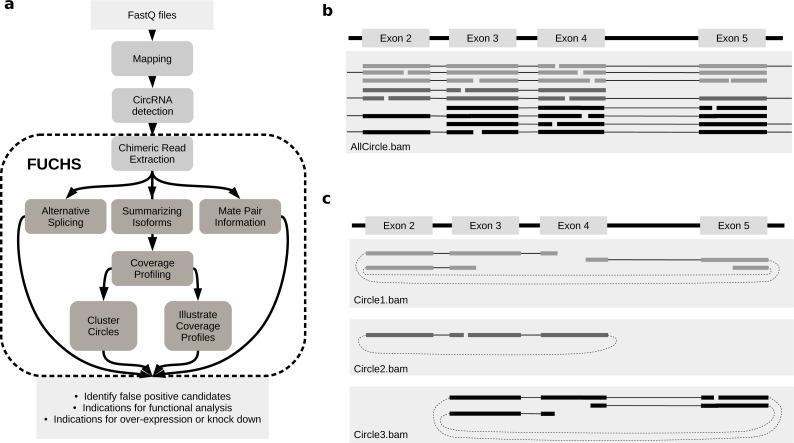
FUCHS workflow and read extraction. (A) FUCHS workflow: first FASTQ files have to be mapped, second, circRNAs have to be detected, these steps are executed by the user before running FUCHS. Running FUCHS will automatically start all steps that are encircled by the dashed line. Most of these steps do not depend on each other and may be skipped with the -sS flag. The output of FUCHS can be used to identify false positive circRNAs. The resulting BED files can be used as input for differential motif enrichment or for miRNA seed analysis to gain knowledge about functionally interesting target circRNAs. (B) All reads: this is a simplified view of a BAM file before circular read extraction. This file may contain linear and chimeric reads, thus being rather cluttered. (C) Reads by circle: separating chimeric reads by circleIDs has the advantage that the structure of these circRNAs is easy to observe, especially when a host-gene harbours many different circular isoforms.

FUCHS is a program designed to run after back-splice junctions have been identified. The purpose of FUCHS is to further describe each circRNA, e.g., identify alternative spliced circles, find double break point fragments, and visualize the coverage profile of a circular RNA. Figure 1A illustrates the work-flow and dependencies of FUCHS.

### CircRNA detection

FUCHS depends on already mapped reads and identified back-spliced junctions. Though it is not in the scope of this work to compare different mapping algorithms or circRNA detection tools, the following section will briefly review current circRNA detection methods.

#### Mapping

To detect circRNAs, first RNAseq reads have to be mapped to the reference genome. There are several mapping programs that are able to map reads to the genome without enforcing co-linearity of query and target. The user may choose one of the following RNAseq mappers: STAR (Dobin et al., 2012), BWA (Li & Durbin, 2010), TopHat-Fusion (Kim & Salzberg, 2011).

#### CircRNA detection

Most circRNA detection tools follow the same strategy but depend on different mappers and employ different filtering steps to rule out false positive candidates. CircRNAs are detected from RNAseq data by finding reads that are not linearly spliced, but where the beginning of a read aligns downstream of the end of the read.

DCC (Cheng, Metge & Dieterich, 2016) requires STAR to map the reads, while CIRI (Gao, Wang & Zhao, 2015) requires BWA mapped SAM-alignments. CIRCexplorer (Zhang et al., 2014) and CIRCfinder (Zhang et al., 2016a) run with TopHat-Fusion mapped reads while KNIFE (Szabo et al., 2015) directly runs with Bowtie2 (Langmead & Salzberg, 2012).

### FUCHS

FUCHS is a tool designed as a Python pipeline to address several questions after circRNA candidates are identified. The following section will discuss the different features of FUCHS.

The pipeline requires only three input files: (1) a tab-separated list of circles, where the first column contains circleIDs and the second column is a comma separated list of names of reads spanning the chimeric junction; (2) a BAM or SAM file containing all chimerically mapped reads, which may also contain linearly mapped and unmapped reads; and (3) a BED formatted annotation file to identify skipped exons and describe the exon-usage. If circRNAs were detected by DCC, the first file is not needed, FUCHS is able to extract all necessary information from the CircRNACount and chimeric.junction.out files.

#### Extracting reads

Visual inspection can be crucial in choosing appropriate circRNAs for further investigation. However, if circular reads overlap with linear or other circular reads, it is not clear how many reads belong to a given circle (Fig. 1B). Therefore, the first step in FUCHS will extract all reads spanning the same chimeric junction and save these reads in separate BAM files resulting in one sorted and indexed BAM file per circleID in the input file.

When opening these BAM files with IGV or other alignment viewers, each circRNA is viewed as a distinct track, as illustrated in Fig. 1C. Hence, it is much clearer which reads belong to which circle. Now it is possible to estimate if a junction is balanced and examine if one or all circles contain an alternatively spliced isoform.

#### Identifying alternative splicing within the same circle boundaries

Alternative splicing is a part of the circular RNA landscape, which cannot be detected by normal circRNA detection programs. Knowing alternatively spliced isoforms is important for an accurate differential motif enrichment as well as miRNA seed analysis. Moreover, with the development of new over-expression or knock down strategies for circular RNA, it is important to know which exons have to be targeted, in order to observe the system’s effect to the candidate circRNA.

**Figure 2:**
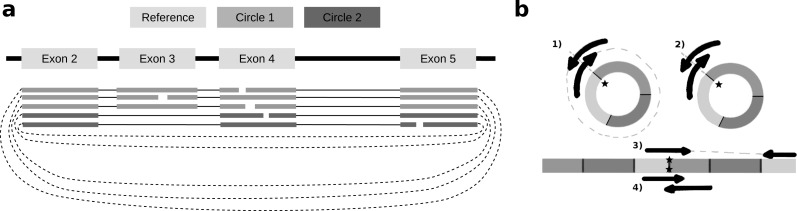
Exon skipping and double-breakpoint fragments. (A) Skipped exons: this schematic view shows two circular isoforms that share the same chimeric junction. With current circRNA detection tools it is not possible to distinguish two isoforms, which share the same chimeric junction. FUCHS is able to distinguish these isoforms. (B) Mate-pair information: this shows the different scenarios on how double-breakpoint fragments emerge from a circular RNA in contrast to a linear RNA. The chimeric junctions are indicated by stars. Double-breakpoint fragments are observed from circular chimeric junctions if the fragment is long enough to capture the whole circle (case 1). The dashed line indicates that the fragment is derived from the whole circular fragment and spanning the chimeric junction twice, whereas the fragment in case 2 is shorter than twice the read length, thus overlapping and spanning the circular junction twice. In contrast, in case 4 the fragment has the same length as in case 2; however, the origin of this junction is a trans-splicing event or genomic rearrangement. Case 2 and 4 are not distinguishable. In case 3, although the fragment is long enough as indicated by the dashed line, the chimeric junction is only covered by one end, because it also originates from a linear transcript.

To identify alternative splice sites within the same circle boundaries, FUCHS first identifies all linear splicing events of all circular reads as illustrated in Fig. 2A. This example has four introns, i.e., between exon 2 and 3, 3 and 4, 4 and 5, as well as 2 and 4. The identified intron boundaries are intersected with annotated exon coordinates using pybedtools (Dale, Pedersen & Quinlan, 2011). Following the example in Fig. 2A, only the intron between exon 2 and 4 overlaps with an annotated exon. Once a skipped exon has been identified, FUCHS intersects the exon coordinates with the circular reads to assign each read to one of the two isoforms. In our example, 2 of 5 reads skip exon 3.

FUCHS reports alternative splicing events in two files. *Sample.skipped_exons.bed* is a BED formatted file containing the information about circle boundaries, intron boundaries and the relative location of the skipped exon, as well as the proportion of reads skipping this exon. This file can be loaded as custom track to any genome browser that accepts BED files. The second file *sample.skipped_exons.txt* additionally lists the read-names of reads skipping the exon, the absolute amount of reads skipping the exon and the amount of total reads. Furthermore, it contains the exact genomic coordinates instead of the relative location, which can be used to extract the genomic sequence of the skipped exon.

#### Mate pair information, indirect validation of circularity

As described in several publications (Abe et al., 2015; You et al., 2015), rolling circles could be observed if a chimeric junction arises from circularized exons. This would not happen if the chimeric junction emerges from a trans-splicing event or genomic rearrangement, thus, indirectly validating that the candidate circRNA is indeed derived from a circularization event.

FUCHS iterates over all reads from one circle and counts how many mate-pairs have only one end spanning the chimeric junction (single, inconclusive) or both ends span the chimeric junction (double, highly likely from circularized exons). Figure 2B illustrates the drawback of this analysis. Case one shows a fragment arising from a circle. The fragment is long enough to capture the full circle resulting in both ends spanning the chimeric junction. In contrast, case 3 shows a fragment from a trans-splicing event. Only one end spans the putative circle junction, although the fragment is also long enough to capture a full circle, thus being a false positive circular RNA junction. Case 2 and 4 pose a scenario in which one cannot distinguish the origin of the fragment. It is too short and the potential circle too long for the fragment to capture the whole circle. The mates overlap in the middle and both circularization as well as trans-splicing could have been the origin of these reads.

This shows the importance of not only quantifying the amount of single- vs. double-breakpoint fragments, but also integrating the actual length of the circular RNA. Thus, considering the circle length as well as the expected fragment size (circle length ≤ fragment length), double-breakpoint fragments can be used to indicate circularity of a given circle.

#### Isoforms from the same host-gene

**Figure 3:**
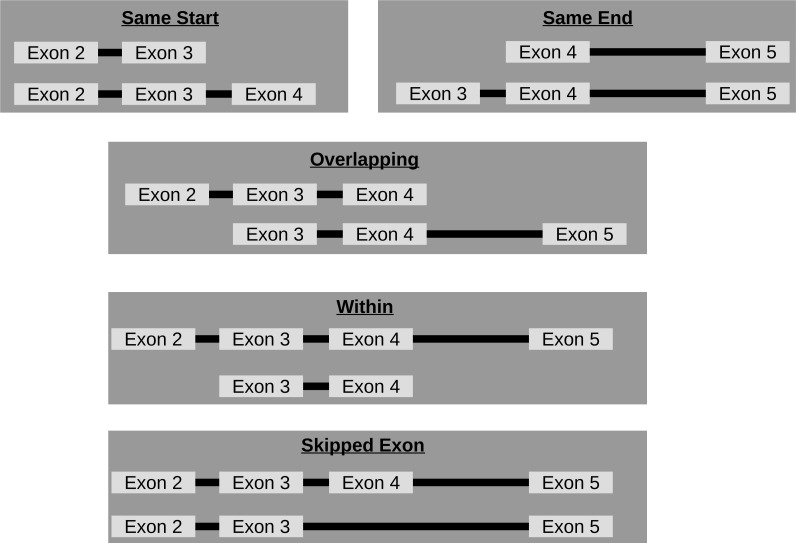
Categories of isoforms: one host-gene can harbour many different circRNAs. This is a schematic view of the different relationships between two circRNAs from the same host-gene.

Though circRNA isoforms arising from the same host-gene that do not share the same start and end location are detected by circRNA detection tools, it is not obvious how many different circles originate from the same host-gene. Therefore, FUCHS summarizes these different isoforms by host-gene and classifies them as same-start, same-end, overlapping, or within (see Fig. 3). This gives the user the opportunity to easily evaluate if any host-gene is prone to giving rise to significantly more circles than the average.

#### Extract coverage

Another characteristic of a circle is the even coverage around the chimeric junction. If there is a bias e.g., an unbalanced chimeric junction, there might be a different origin for the chimeric junction than circularization. Using pybedtools coverage, FUCHS generates an exon-wise coverage profile for each circle. The obtained information is saved in two files. First, a table in BED format, in which each row corresponds to a circle, containing the information about covered exons. This file can be loaded into any genome browser. The second, more detailed table, lists the coverage information per exon, as well as its length.

Additionally, FUCHS generates a position-wise coverage profile for each circle. The output file is a tab-separated file, containing the information of each base’s exon number, its relative position within the circle, its relative position within the exon, and its coverage.

Based on the exon-count table and coverage profiles, FUCHS is able to proceed with several optional characterizations of the circles summarized in the following section.

#### Optional steps based on coverage profiles

Using R, FUCHS will generate a graphical representation of each circle’s coverage profile, preserving the exon information as coloured segments. The smoothed profiles are saved as PNGs in a separate folder for easy examination by eye.

Furthermore, FUCHS will accumulate all coverage profiles, normalize the profiles by circle length and cluster the circles based on their coverage profiles. The clustering is performed on all circles. Additionally, to avoid that the clustering will only group the circles based on their length, a group-wise clustering is performed. Here the circles are separated based on their length into small (<500 BP), medium (500–1,000 BP), and long (≥1,000 BP) circles. Based on correlation a K-means clustering is performed using the R package *amap*. The number of clusters is determined by the number of circRNAs in each group, i.e., small, medium, long, and all. For sets that contain only two circRNAs no clustering is performed. For sets of three to nine circRNAs, two initial points are chosen. For sets of 10–100 circRNAs, four initial points are chosen and for sets of more than 100 circRNAs the number of clusters is determined by $round( \frac{\sharp \mathrm{circRNAs}}{20} )$ but never more than 10 clustres are choosen. Based on these clustering results we hope to identify false positive circRNA candidates by screening for cluster that show a clear uneven coverage around the circular junction and not a bell curve around the chimeric junction (compare).

### Data

We tested FUCHS on human HEK293 rRNA depleted samples as well as heart and liver rRNA depleted and RNAse-R treated (circSeq) samples of C57BL/6 mice. Two biological replicates were sequenced for each condition. The HEK293 data set was generated on an Illumina MiSeq system with 2 × 300 BP reads and the heart and liver samples were generated on an Illumina HiSeq2500 with 2 × 250 BP reads respectively. We have made all samples available. All HEK293 RNA-seq data sets are available via SRA study SRP050149. All murine RNA-seq data sets are available via SRA study SRP097141.

**Table:** 

Sample	Species	Library-prep	Reference genome	SRA experiment accession
HEK293.1	HEK293	rRNA depleted	hg38	SRX2431511
HEK293.2	HEK293	rRNA depleted	hg38	SRX2473824
Liver.1	Black-6 mouse	rRNA depleted/RNaseR+	mm10	SRX2504988
Liver.2	Black-6 mouse	rRNA depleted/RNaseR+	mm10	SRX2504990
Heart.1	Black-6 mouse	rRNA depleted/RNaseR+	mm10	SRX2504987
Heart.2	Black-6 mouse	rRNA depleted/RNaseR+	mm10	SRX2504989

The libraries were prepared using the ScriptSeq v2 RNA-Seq Library Preparation Kit from Illumina (epicentre, an Illumina company), which involves a random RNA fragmentation step before cDNA synthesis. Thus, longer circRNAs have a higher probability of being broken up before cDNA synthesis, but generally some circRNAs may stay intact and could be subject to rolling circle PCR amplification.

Only STARlong is able to map reads that are longer than 249 base pairs for one end, but because STARlong is not capable of mapping chimeric reads, the reads needed to be mapped using STARshort. On this account all reads were trimmed to 249 base pairs using Flexbar version 2.5 (Dodt et al., 2012) with the following parameters.

 
 
         Listing 1: FlexBar 
flexbar −n 4 −r  sample_R1.fastq.gz −p  sample_R2.fastq.gz 
−t sample −f sanger −u 5 −k 249 −z GZ    

All RNA-seq data were mapped using STARshort version 2.5.1b to the human genome hg38 or the mouse genome, mm10 respectively.

 
 
                          Listing 2: STAR 
STAR −−outSJfilterOverhangMin  15 15 15 15 
 −−alignSJoverhangMin  15 
 −−alignSJDBoverhangMin  10 
 −−outFilterMultimapNmax  20 
 −−quantMode  GeneCounts 
 −−outFilterMismatchNmax  999 
 −−outFilterMatchNminOverLread  0.7 
 −−alignMatesGapMax  1000000 
 −−outFilterMismatchNoverLmax  0.05 
 −−outFilterScoreMin 1 
 −−alignIntronMin  20 
 −−alignIntronMax  1000000 
 −−chimSegmentMin  15 
 −−chimScoreMin  15 
 −−chimScoreSeparation  10 
 −−chimJunctionOverhangMin  15    

CircRNA candidates were detected by back-splicing junction searches using DCC version 0.3.2 with annotation from Ensembl build 79.

 
 
                          Listing 3: DCC 
DCC  Chimeric.out.junction −ss −D −an  annotation.gtf 
−R repeats.gtf −Pi −mt1  mate1.Chimeric.out.junction 
−mt2  mate2.Chimeric.out.junction −A reference.fa 
−B Aligned.bam −M −Nr 2 1 −fg −G −temp −F −L 20    

## Results and Discussion

### Isoforms

Summarizing the different circles emerging from one host-gene only 20 percent of host-genes in HEK293 and 50 percent of host-genes in mice exhibit more than one circle. The most prominent multiple circles have either the same start or end coordinates. This observation supports previously reported regulatory elements in introns neighbouring circularized exons. Overlapping circles or circles completely contained in another circle’s boundaries are less prominent isoforms.

**Figure 4:**
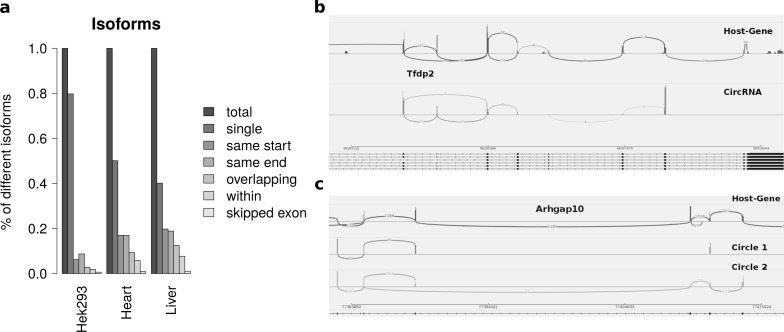
Results from summarizing circRNAs by host-gene and detecting alternative splicing within the same circle boundaries. (A) Distribution of isoforms: this shows the proportion of host-genes harbouring only one circle (single) or several different circle. These circles are classified into different categories as shown in Fig. 3 (B) Alternative splicing of circTfdp2: this is an example of two isoforms with the same circleID. Both splice variants are annotated and present in the host-gene. (C) Alternative splicing of circArhgap10: in contrast to b, circle 2 of Arhgap10 shows a new splicing variant that is neither annotated nor present in the host-gene or circle 1.

While most alternatively spliced circular isoforms are present as annotated linear isoform (Fig. 4B), there are also few circRNAs in which a novel alternative splicing occurs (Fig. 4C). Here the third exon is skipped in one of the circRNA isoforms. This additional isoform is neither annotated nor present in the host-gene or other circular isoforms spanning this exon.

### Circle length and double-breakpoint fragments

**Figure 5:**
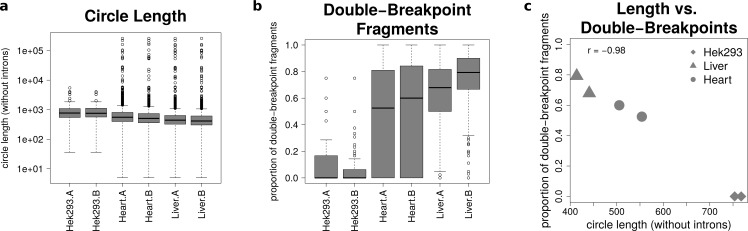
Results from analysing the mate-pair information. (A) Circle length: this boxplot shows the distribution of absolute circle length of all circRNAs per sample. (B) Double-breakpoint fragments: this boxplot shows the distribution of the relative amount of double-breakpoint fragments over all fragments per circle per sample. Intruigingly the distribution is broader in mouse hearts than mouse liver. (C) Length vs. double-breakpoints: this scatteplot summarizes the previous plots. Only the median proportion of double-breakpoint fragments in dependence of the median circle length is shown for each circle. The propotion of double-breakpoint fragments is negatively correlated with the circle length (*r* =  − 0.98).

The analysis of mate-pairs spanning the chimeric junction yields not only information about the fraction of double-breakpoint fragments but also about the estimated length of circRNAs. Figure 5A shows that there is no significant difference in the length of circRNAs between the replicates of the human samples (*p* = 0.89), whereas human circRNAs are significantly longer than circRNAs detected in both mice tissues (*p* = 9.36∗10^−36^). The median circle length ranges from 750 base pairs in the HEK293 samples to 430 base pairs in the mouse liver.

Figure 5B shows that while HEK293 has only a few double-breakpoint fragments, the mouse liver has the highest proportion of double-breakpoint fragments. Intriguingly the distribution of the relative amount of double-breakpoint fragments from mouse circRNAs in the heart is broader then of the other samples.

Correlating the median circle length with the median proportion of double-breakpoint reads a strong negative correlation (*r* =  − 0.98, Fig. 5C) between the length and the fraction of double-breakpoint fragments becomes evident. This indicates that double-breakpoint fragments can be used to identify false positive circRNA candidates. In circles that are smaller than the read length, we observe that the end of the read has the same sequence as the beginning of the read, hence the read must have originated from a circular RNA that was not fragmented during the library preparation step. Circles shorter than 500 base pairs are small enough to be fully captured by circSeq, often both ends of the mate-pairs are spanning the chimeric junction if the origin is a circular RNA. With increasing circle size, it becomes impossible for both ends two span the chimeric junction, unless the fragment is shorter than twice the read length, thus both ends overlap over the chimeric junction.

### Clustering of circRNAs

**Figure 6:**
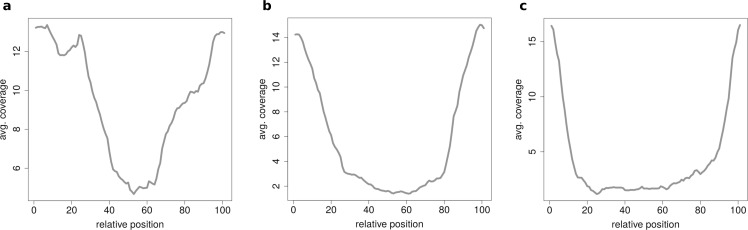
Results of the clustering analysis in HEK293.B. These plots show the coverage profile averaged over all circles belonging to each cluster. The major clusters separate the circle by their circle length. (A) Typical profile for shorter circles. The median length in this cluster is 560 base pairs. (B) Typical profile for medium circles. The median length in this cluster is 1,073 base pairs. (C) Typical profile for longer circles. The median length in this cluster is 1,434 base pairs.

When clustering circRNAs based on their coverage profiles, it becomes noticeable that different length of circRNA have distinguishable coverage profiles. If the clustering is performed on all circRNAs, the circRNAs will be grouped according to their length. Figure 6A shows an expected profile of short circRNAs, the median length of circles belonging to this cluster is only 560 base pairs whereas circRNAs from cluster in Fig. 6B are of 1,073 base pairs median length. Very long circRNAs are only covered around the chimeric junction and no reads align to the middle of the circle. This profile can be observed in Fig. 6C where circRNAs are 1,434 base pairs long on average.

**Figure 7:**
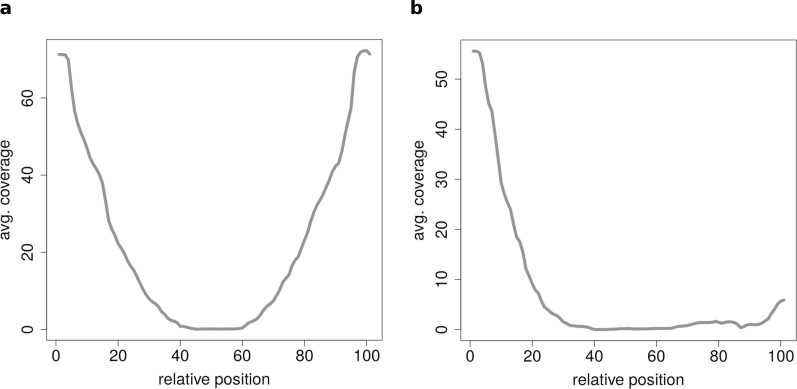
Results of the clustering analysis of only medium length circles (500–1,000 BP) in mouse liver B. (A) Average coverage cluster 1: the biggest cluster contains 64 members and shows the typical coverage profile of medium length circle (compare Fig. 6B). (B) Average coverage cluster 2: this cluster has 22 members, in contrast to cluster 1 it shows an uneven coverage profile. This could indicate that the chimeric junctions originated from a different event other than circularization of exons.

If circRNAs are grouped by length (short, medium, long) before the clustering analysis is performed unexpected coverage profiles emerge. While the majority of cluster show a for this length typical coverage profile such as Fig. 7A, a few cluster contain circRNAs with an unusual coverage profile such as an unbalanced chimeric junction as in Fig. 7B. While circRNAs with an even coverage profile are likely to be true circRNAs (compare Fig. 2B case 1 and 2), candidates with an unbalanced junction could originate from other events (compare Fig. 2B case 3).

## Conclusion

This study presents a new tool to help further characterize circRNA candidates. Our tool, FUCHS, is able to identify alternative exon usage within the same circle boundaries, summarize the different circles emerging from the same host-gene, quantify double-breakpoint fragments as indicator for circularity and visualize a circRNA’s read coverage profile independent of any genome browser.

When over-expressing circular isoforms to gain insights into the function or biogenesis of the target circRNA it is crucial to know the exact exon structure in order to express only the desired isoform. Furthermore, integrating knowledge about the exact exon structure could lead to a more accurate differential motif enrichment and miRNA seed analysis. Information about double-breakpoint fragments and coverage profiles of a circRNA can be used to filter out likely false positives e.g., if a circRNA is supported only by single-breakpoint fragments, although the circle is shorter than the fragment length, thus if it was a true circRNA it should be supported by double-breakpoint fragments. Plotting the individual coverage profiles allows the user to quickly examine all circles to get a general idea about coverage, length and number of exons, as a first quality measure of the data.

Altogether FUCHS is a valuable tool to characterize circRNAs. FUCHS provides the user with directions for further steps to investigate the circRNA’s function and biogenesis.

## Additional Information and Declarations

### Data Deposition

The following information was supplied regarding data availability: GitHub: https://github.com/dieterich-lab/FUCHS/tree/master/GCB_testset. We have made all samples available. All HEK293 RNA-seq data sets are available via SRA study SRP050149). All murine RNA-seq data sets are available via SRA study SRP097141.
